# A Comparative Study of Quality of Life and Oncologic Outcomes in Premenopausal Women with Hormone Receptor-Positive Breast Cancer: Bilateral Oophorectomy vs. Gonadotropin-Releasing Hormone Agonist Therapy

**DOI:** 10.3390/cancers17172916

**Published:** 2025-09-05

**Authors:** Evrim Erdemoglu, Kathryn J. Ruddy, Matthew R. Buras, Jaxon Quillen, Fergus J. Couch, Janet E. Olson, Laura M. Bozzuto, Nicole L. Larson, Johnny Yi, Kristina A. Butler

**Affiliations:** 1 Department of Medical and Surgical Gynecology, Mayo Clinic, Phoenix, AZ 85054, USA; evrimmd@yahoo.com (E.E.); yi.johnny@mayo.edu (J.Y.); 2 Division of Gynecologic Oncology, Department of Obstetrics and Gynecology, Suleyman Demirel University, Isparta 32260, Turkey; 3 Department of Oncology, Mayo Clinic, Rochester, MN 55905, USA; ruddy.kathryn@mayo.edu; 4 Division of Clinical Trials and Biostatistics Quantitative Health Science Research, Mayo Clinic, Arizona, MN 55905, USA; buras.matthew@mayo.edu (M.R.B.); quillen.jaxon@mayo.edu (J.Q.); 5 Department of Laboratory Medicine and Pathology, Mayo Clinic, Rochester, MN 55905, USA; couch.fergus@mayo.edu; 6 Department of Quantitative Health Science, Mayo Clinic, Rochester, MN 55905, USA; olsonj@mayo.edu (J.E.O.); larson.nicole2@mayo.edu (N.L.L.); 7 Division of Surgical Oncology, Department of Surgery, Wisconsin School of Medicine and Public Health, Madison, WI 53705, USA; bozzuto@wisc.edu

**Keywords:** breast cancer, quality of life, oophorectomy, gonadotropin-releasing hormone, GnRH, ovarian function suppression, PROMIS-10, patient-reported outcomes

## Abstract

**Background/Objectives:** This study aims to evaluate the quality of life (QoL) and oncological outcomes in premenopausal women diagnosed with hormone receptor-positive breast cancer who are receiving either bilateral oophorectomy (BO) or gonadotropin-releasing hormone agonist (GnRH) therapy. Both methods serve to inhibit ovarian function, which is essential for the management of estrogen-dependent tumors; however, their effects on QoL have yet to be fully clarified. **Methods:** Data were analyzed from the Mayo Clinic Breast Disease Registry, focusing on women under 55 diagnosed with estrogen receptor-positive breast cancer who received either BO or GnRH within one year of diagnosis. QoL was assessed using the Patient-Reported Outcomes Measurement Information System Global-10 (PROMIS-10) at baseline and annually for five years. **Results:** A total of 181 patients were enrolled in the study; 40 into the BO group and 141 to the GnRH group. Both groups exhibited similar levels of sexual dysfunction after a one-year period; however, the BO group stated a higher frequency of hot flashes. PROMIS-10 scores improved in both mental and physical health over time, with no significant differences between the groups. Within the BO group, one recurrence was observed, in contrast to the GnRH group, which had six events. Nonetheless, the difference in recurrence rates did not reach statistical significance. **Conclusions:** The long-term QoL and oncologic outcomes for premenopausal women with hormone receptor-positive breast cancer were similar for BO and GnRH therapy. These findings emphasize the need for individualized treatment decisions, considering patient preferences and side effects.

## Simple Summary

Ovarian suppression to diminish estrogen levels to prevent cancer recurrence is usually required in premenopausal women with hormone receptor-positive breast cancer. This can be achieved either surgically through bilateral salpingo-oophorectomy (BO) or medically using gonadotropin-releasing hormone agonists (GnRH). Although both approaches are commonplace, there is less knowledge about their impact on long-term quality of life, particularly in women with BRCA mutation negative breast cancer. This study aimed to compare physical, emotional, and functional well-being across the two strategies, utilizing a validated patient-reported outcome measure. We conducted a prospective longitudinal analysis of 181 women with breast cancer who underwent BO or received GnRH. During follow-up of five years, we observed no significant differences in overall quality of life, mental health, or physical function between the two cohorts. Patients in the BO cohort reported a higher frequency of hot flashes. The rates of cancer recurrence and disease-free survival were comparable. Our findings indicate that both surgical and medical ovarian suppression are feasible alternatives with similar long-term outcomes. Assisting individuals in selecting options for ovarian suppression that correspond with their values and interests can enhance both survivorship and patient satisfaction.

## 1. Introduction

The majority of breast cancers are estrogen-dependent, and the suppression of ovarian function by different methods is an important treatment for premenopausal breast cancer management [1,2,3,4]. Premenopausal women with estrogen receptor-positive tumors are less likely to experience recurrence and are treated with gonadotropin-releasing hormone agonist (GnRH) or bilateral oophorectomy (BO), both forms of ovarian function suppression (OFS) [1,2,3]. In 2003, the International Breast Cancer Study Group (IBCSG) initiated two impactful randomized phase 3 trials: the Suppression of Ovarian Function Trial (SOFT) and the Tamoxifen and Exemestane Trial (TEXT), both enrolling premenopausal women with hormone-receptor-positive early breast cancer. SOFT aimed to evaluate the benefits of incorporating ovarian suppression with tamoxifen or the aromatase inhibitor exemestane (compared to tamoxifen without ovarian suppression). OFS was achieved by BO, radiotherapy, or triptorelin (intramuscular GnRH). The results after 8 years showed significant improvements in disease-free survival and overall survival (OS) with the addition of OFS to tamoxifen, and even larger improvements when exemestane was given in combination with OFS [2]. Subsequent guidelines recommended the use of OFS in premenopausal women at heightened risk of recurrence.

Menopausal side effects, sexual dysfunction, and quality of life (QoL) are critical concerns for young women undergoing OFS. Hot flashes, vaginal dryness, and other side effects may contribute to endocrine therapy nonadherence, which has been identified in up to 20% of patients [4,5]. For that reason, most clinicians recommend that patients receive GnRH for a period before undergoing BO. Subsequently, the decision to proceed with BO versus to continue GnRH can be a difficult one. While medical suppression is reversible, it requires frequent clinical visits and lab tests, and it carries a risk of incomplete menstrual suppression. And patients with a cancer diagnosis may have a strong negative aversion to frequent medical visits [6]. Surgical menopause can be performed through minimally invasive techniques, typically laparoscopy, and has no risk of failure. However, oophorectomy is permanent (does not allow return of ovarian function later), which may adversely impact cardiac, bone, and cognitive health [7]. In SOFT and TEXT, OFS was achieved through medical agents, surgery or irradiation. However, QoL was not compared directly compared between oophorectomy and others [2,5,8,9,10,11,12]. Currently, there is no clear evidence determining whether surgical menopause or medical suppression is superior for premenopausal breast cancer treatment, either for cancer-related outcomes or for QoL [9,10,11,12].

Given the lack of clear evidence on whether BO or GnRH is superior, our objective was to compare QoLand cancer outcomes between BO and GnRH amongst breast cancer survivors.

## 2. Methods

This study analyzed data from the Mayo Clinic Breast Disease Registry (MCBDR), which is a prospective longitudinal cohort of patients enrolled within a year of an initial breast cancer diagnosis who agree to the collection of clinical, pathological, and patient-reported outcomes (PROs) pertinent to breast cancer diagnosis, treatment, and survivorship. Data for this analysis were accessed on 30 May 2024. Informed consent is required, and all enrollees must have been seen at least once at Mayo Clinic Rochester for their breast cancer. Participants were asked to complete an electronic or paper baseline survey at the time of enrollment as well as annual follow-up surveys (for which the average response rate was 65%). The baseline and year 1–5 follow-up surveys were utilized. Medical record abstraction was used to understand treatments received and tumor characteristics.

**Figure 1:**
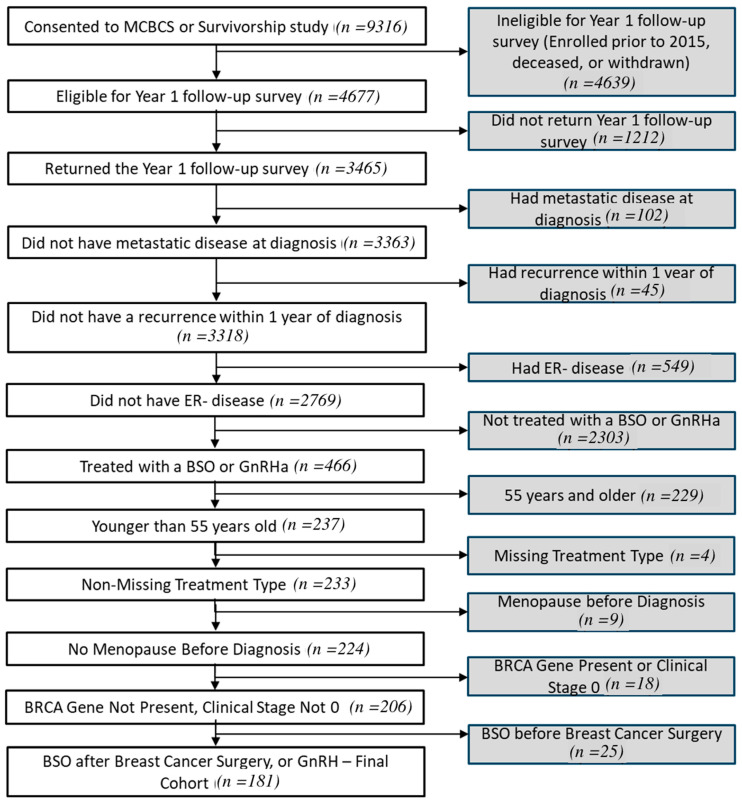
Flowchart of participant.

Women under age 55 with a diagnosis of estrogen receptor-positive breast cancer treated with BO and/or GnRH within 1 year after diagnosis of breast cancer were included in this analysis. The criteria for exclusion were as follows: known deleterious *BRCA1/2* mutation, prior oophorectomy, and/or GnRH use before the diagnosis of breast cancer, or missing year 1 survey data. Patients with deleterious BRCA mutations were excluded as this would prompt oophorectomy according to NCCN risk reducing guidelines. Further data exclusions were included to identify and exclude patients with stage 0 disease and those missing treatment type. The flow diagram is shown in Figure 1. The MCBDR was approved by the Mayo Clinic Institutional Review Board (IRB# 20-012602). This research complies with the STROBE (Strengthening the Reporting of Observational Studies in Epidemiology) guidelines for cohort studies. The OFS method was defined as BO if a bilateral oophorectomy was performed between 0 and 12 months after the breast cancer diagnosis (even if GnRH was also administered prior to that). Otherwise, the OFS method was defined as GnRH if there was at least one dose of GnRH administered between 0 and 12 months after the breast cancer diagnosis, with no BO during that period. If a BO was performed after the 12-month timepoint, the patient OFS method was still categorized as GnRH.

### 2.1. QoL Assessments

The Patient-Reported Outcomes Measurement Information System Global-10 (PROMIS-10) was utilized to evaluate the QoLat baseline and annually in follow-up surveys. PROMIS-10 is a validated and extensively employed instrument for measuring overall health across diverse patient populations [13,14,15,16]. This instrument (PROMIS; see www.nihpromis.org (accessed on 10 August 2025) yields two summary scores: The Global Physical Health (GPH) Score and The Global Mental Health (GMH) Score. GPH measures physical functioning, fatigue, pain, and overall health perceptions. GMH Score evaluates emotional well-being, social engagement, and psychological resilience [13].

QoL surveys (Supplementary material) conducted prior to treatment and 1, 2, 3, 4, and 5 years following treatment were analyzed. All surveys were conducted through self-administration, utilizing either paper or electronic formats, and were subsequently documented within the electronic health record system. Alongside PROMIS-10, the investigation incorporated domain-specific assessments of QoL, concentrating on the severity of fatigue, emotional well-being, participation in social activities, satisfaction in relationships, and interference due to pain. Results were presented as patient characteristics, global health and PROMIS-10 scores, domain-specific QoLanalysis.

Patients were asked at baseline and follow-up at 1 year to “check the number (0 to 10) that best reflects your concerns and symptoms”, including their “sexual dysfunction” and “hot flashes”, rated on numeric rating scales (NRS) ranging from 0 (“none”) to 10 (“the worst you can imagine”).

### 2.2. Data Acquisition and Management

Data collection was performed according to a standardized protocol that was developed as part of the Mayo Breast Disease Registry. Enrolled patients were required to complete an initial baseline QoLsurvey, followed by follow-up surveys administered at predetermined intervals. Individuals who did not submit their questionnaires received reminder messages every two weeks up to a total of three reminders.

### 2.3. Statistical Analyses

Descriptive statistics for categorical data are summarized as count and percent while numerical data are summarized with the median and interquartile range (IQR). Comparisons of the distributions of categorical variables between groups were conducted with Fisher’s Exact test, while distributions of numerical variables were compared using the Kruskal–Wallis test (Table 1). If an item-response needed to calculate a score was missing, the participant was then said to have a missing score as well.

**Figure 2:**
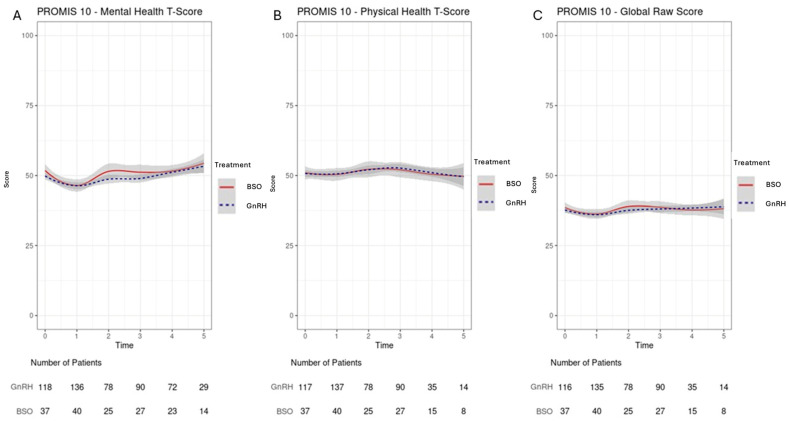
The confidence intervals of GnRH and BO group converged, with no significant difference between groups for Mental Health Score, Physical Score, and Global Raw Score. (**A**) The PROMIS 10 Mental health t score declined at 1 year and restored to the baseline by 0.5 increase per year (*p* = 0.0002); (**B**) the PROMIS 10 Physical Health Score, although there was a slight improvement by year 2–3, returned to baseline by year 5. This slight change is statistically significant, but it is clinically not; and (**C**) global raw score physical health scores. Time is given in years. Global raw scores increased slightly over time (0.3 points/year, *p* = 0.017).

**Figure 3:**
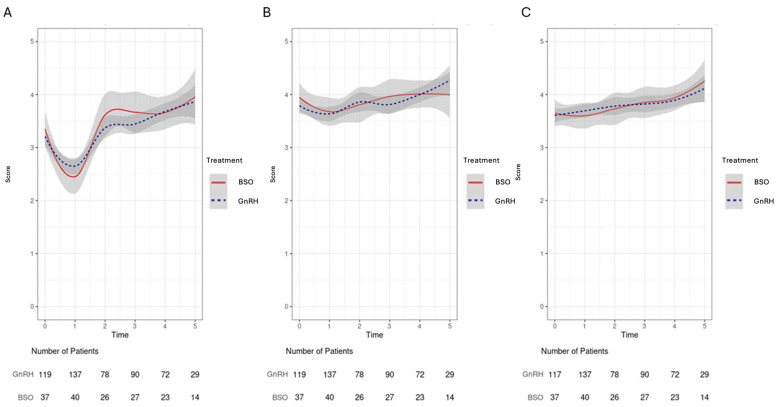
The confidence intervals of GnRH and BO group converged, with no significant difference between groups. (**A**) Emotional well-being dipped at year 1, then improved steadily, reaching higher than baseline by year 5 (0.2 points/year, *p* < 0.0001). (**B**) Social activity scores remained stable with modest late improvement. (**C**) Fatigue scores improved gradually (0.1 points/year, *p* < 0.0001); moderate fatigue decreased from 30.8% to 7% over 5 years. The red-solid line represents those in the BSO group while the blue-dotted line represents those in the GnRH group.

Each PROMIS question was modeled as a numeric outcome with a range of 0–5. Each calculated PROMIS score (e.g., mental health t-score) are plotted on a scale of 0–100. A linear regression model with a random intercept was used to quantify the differences in PROMIS-10 scores between treatment groups and over time. This modeling procedure allows a varying number of observations per participant wherein only non-missing scores are included on a timepoint-by-timepoint basis. This allows the inclusion of participants with missing scores to be included in the model as well. The statistical interaction between time and treatment group was not statistically significant at the 0.05 level for all models and was therefore excluded from all models discussed in this paper. Plots of trend for PROMIS-10 scores for each group over time were created using the LOESS smoother (R v4.3.2 gplot2) (Figure 2 and Figure 3).

Survival curves for time to recurrence from date of diagnosis were calculated using the Kaplan–Meier method. The log-rank test was used to test for a statistically significant separation of survival curves.

All hypothesis tests were two-sided with *p* < 0.05 considered statistically significant. All analyses were performed in R v4.3.2. (R Core Team 2023).

## 3. Results

### 3.1. Patient Characteristics

A total of 181 patients were included in this analysis. There were 40 patients in the BO group and 141 patients in the GnRH group. Table 1 presents their demographics and oncological characteristics. Patients in the BO group were older (median (IQR) 46 (42.9–48.3) vs. 39 (33.9–46.9), *p* < 0.001) and had lower stage disease (55% Stage I in BO vs. 26% in GnRH, *p* < 0.001) compared to patients in the GnRH group (*p* = 0.002). Other clinical and oncological characteristics were comparable among the groups.

**Table 1:** Demographic and oncological features of the participants.

	BO (*n* = 40)	GnRH (*n* = 141)	*p* Value
Age at diagnosis (median-IQR)	46 (42.9–48.3)	39.8 (33.9, 46.9)	<0.001
Vital status (Alive) at 5-y	95%	97.9%	0.30
Race			0.64
White	97.5%	92.9%	
Black/African American	-	0.7%	
Asian	-	4.3%	
Other *	2.5%	2.1%	
Ethnicity (non-Hispanic)	97.5%	92.9%	1.0
Overall Stage			<0.001
Stage I	55.0%	26.2%	
Stage II	37.5%	50.4%	
Stage III	7.5%	23.4%	
Surgery Type			0.07
Lumpectomy and Mastectomy	0	5%	
Lumpectomy	37%	14.9%	
Mastectomy and No Lumpectomy	52.5%	72,3%	
No Mastectomy and No Lumpectomy	10%	7.8%	
ER Status (Positive)	100%	99.3%	1.0
PR Status (Positive)	100%	90.6%	0.11
HER2 Status (Negative)	87.2%	82.7%	0.32
Recurrence (No)	97.2%	95.3%	1.0
Baseline (Median-IQR)			
Hot Flashes	0.0 (0.0, 5.0)	1.0 (0.0, 5.0)	0.49
Sexual Dysfunction	0.0 (0.0, 5.0)	1.0 (0.0, 3.8)	0.75
At Year 1			
Hot Flashes	6.0 (2.0, 7.2)	4.0 (2.0, 6.0)	0.02
Sexual Dysfunction	4.0 (1.0, 6.0)	3.0 (1.0, 6.0)	0.45
Time between diagnosis and last survey	4.9 (2.2, 7.1)	4.0 (2.2, 5.9)	0.19
Time between diagnosis and Last vital status	6.0 (3.7, 8.4)	5.3 (3.5, 7.3)	0.27

* Race other includes American Indian/Alaskan native and choose not to disclose. IQR: Interquartile range Q3-Q1 designating 50% of data.

### 3.2. Side Effects

Sexual dysfunction was similar between the groups at 1 year, but patients in the BO group reported more hot flashes compared to patients in the GnRH group (Median 6 vs. 4, *p* = 0.02)

### 3.3. QoLMeasures

Average time between diagnosis and last survey was similar in both groups (median (IQR) 4.9 (2.2–7.1) years in BO group vs. 4.0 (2.2–5.9) years in GnRH group, *p* = 0.19).

Baseline PROMIS-10 scores were similar across the groups (Table S1. The global raw PROMIS-10 score increased regardless of treatment 0.3 per year (95CI%:0.0–0.5, *p* = 0.017). The PROMIS-10 Mental health t-score declined at 1 year, followed by 0.5 per year increase on average in both groups, (95%CI: 0.2–0.8, *p* = 0.0002) and returned to baseline value by year 5 (Figure 2). The PROMIS-10 physical health score increased regardless of treatment by 0.3 per year on average (95%CI:0–0.6, *p* = 0.03); however, this slight change was not clinically meaningful at year 5 (Figure 2B). In both groups, the increase in physical score was most evident at year 2–3 but returned back to baseline by year 5 (Figure 2).

### 3.4. Domain-Specific QoLAnalysis: Emotional Well-Being/Fatigue/Social Activity/Relationship Satisfaction

The proportion of patients in both groups are shown in Figure 3. The emotional well-being scores were similar at baseline, and there were improvements at Y1–Y2 and then at Y3. Emotional well-being averaged 0.2 increase per year from baseline to Y5 in both groups (95%CI: 0.1–0.2, *p* < 0.0001), with no significant differences in these changes over time between the groups (*p* = 0.59), shown in Figure 3.

Fatigue scores improved over time and did not differ by type of OFS (Figure 3). Fatigue scores showed 0.1 per year improvements per year (95%CI: 0–0.1, *p* < 0.0001). Moderate fatigue decreased from 30.8% to 7%, and the proportion reporting “None” increased from 17.3% to 34.9% over the 5-year follow-up (*p* = 0.036) (Supplementary Table S2).

Social health improved over time in the overall cohort (95%CI: 0–0.1, *p* = 0.003). Although there were no statistically significant differences between the BO and GnRH groups with regard to changes in social health over time, there were numerically greater improvements evident in the GnRH group. In the BO group, 78% of patients initially reported that their social health was “very good” or “excellent,” and this changed to 85% by five years. In the GnRH group, those reporting “very good” or “excellent” social health increased from 68% to 92%. Relationship health was assessed and did not differ by group at 1 year or over time (*p* > 0.05). Adjusting PROMIS-10 individual and composite scores for age and overall clinical stage (I, II, III) resulted in no statistically significant differences between treatment groups. Results of these analyses are found in the supplement (Table S2).

### 3.5. Oncological Outcomes

There was 1 recurrence in the BO group (2.5%) and 6 in the GnRH group (4.3%) (log-rank test, *p* = 0.52). The survival curve is shown in Figure S1.

## 4. Discussion

Assessing the effects of OFS on QoL in women with breast cancer and no known BRCA mutation may inform decisions about premenopausal breast cancer therapy. Although we had hypothesized that oophorectomy might be associated with more menopausal symptoms and poorer QoL than GnRH, we found no difference between the groups. Our findings included increasing hot flashes, vaginal dryness in both groups over time; however, the intensity of the hot flashes was more intense in the BO group. Improvements in mental health, fatigue, emotional well-being, and social activity were also observed. Other domains of QoL were stable over time. For oncological outcomes, limitations of our study included that there were too few recurrences in this cohort to confirm equivalent oncologic outcomes. Although the follow-up time was similar between the two cohorts (4.9 year vs. 4 year), this relatively short follow-up may have limited our ability to detect differences in risk of recurrence. Further, long-term morbidity was not assessed after 5 years, and we did not have access to measures of cardiovascular or bone health, which could differ between these groups.

In a previous report from Sloan-Kettering, like in our study, older patients were more likely to choose oophorectomy over GnRH in the absence of a BRCA mutation [12]. This may be due to concerns by younger women and their doctors about the potentially detrimental cardiovascular, cognitive, bone, and/or other side effects of permanent OFS long before the age of natural menopause. In addition, fertility concerns may play a role if a younger woman is considering future childbearing such that a temporary approach to OFS is more appealing. Our finding of more early-stage disease in the BO group than in the GnRH group requires additional investigation, as we had hypothesized that patients with higher stage disease (which has a greater risk of recurrence) might be more likely to opt for an irreversible OFS approach.

QoL in women with breast cancer may be influenced by factors including age, stage, socioeconomic status, body image, and fear of recurrent cancer [17,18]. These factors have a complex inter-relationship [19] and might also impact female sexual function. Despite the difference in age and breast cancer stage, no difference was observed in female sexual function parameters between the treatment groups. Consistent with our findings, in a previous cross-sectional study, sexual dysfunction was similar with or without oophorectomy in breast cancer survivors [20]. However, we found that the intensity of hot flashes was significantly greater in the BO cohort in comparison to the GnRH cohort. The increased intensity of hot flashes observed in patients who have undergone BO may be due to a more complete suppression of ovarian function with this approach. In the SOFT-EST sub-study, at least 17% of patients on GnRH had estrogen levels greater than the postmenopausal threshold [21]. The 2007 TABLE Study by Schmid et al. noted a hormonal escape rate of 10.4% (defined as estradiol levels greater than 30 pg/mL at two consecutive measurements) in the leuprorelin acetate in the 3-month interval group [22].

Mental health is an important consideration for cancer survivors. The mental health scores in PROMIS-10 showed improvement in both the GnRH and BO treatment groups, with an average increase of 0.5 per year. Over five years, this 2.5-point average increase is above the threshold for clinical relevance and should be reassuring to patients and clinicians. However, this and other studies have observed that long-standing physical symptoms including musculoskeletal pain and fatigue are common during OFS [23,24,25]. The development and implementation of interventions that address physical health concerns are important to prevent patients from prematurely discontinuing endocrine therapy, as that may decrease the effectiveness of treatment [19,26,27]. Targeting exercise regimens and physical therapy to those at highest risk of toxicities may help to optimize the health of premenopausal breast cancer survivors [19,28,29].

It is reassuring that fatigue and emotional well-being were both significantly improved in both treatment groups. While fatigue is a common complaint in the general population, it has special relevant causes and importance in patients with breast cancer [30,31,32,33]. It has been suggested that fatigue can be related to the dysfunction of the hypothalamic–pituitary–adrenal axis, cytokines, alterations in circadian rhythms, dysregulation of 5-hydroxy tryptophan impacting QoL in breast cancer survivors [33]. Incidence of daily fatigue in cancer survivor patients ranges from 17% to 30% and can profoundly affect QoL [33,34]. Fatigue has been associated with reductions in mental, psychological, physical, and social functioning, which are critical domains of QoL [35,36]. Many individuals have indicated that fatigue is the most troubling symptom they have encountered, surpassing even pain in this regard [34,37].

Prior research has shown that social integration is a key determinant of overall well-being in breast cancer survivors [38]. Interventions intended to encourage return-to-work, peer support, and family counseling have the potential to improve these outcomes [39]. Our analysis revealed a positive trajectory in social activity and relationship satisfaction, with no significant differences observed between the BO and GnRH groups. Healthcare professionals must consider these findings when advising patients, as the preservation of social functioning may represent a significant priority in treatment strategies. The strengths of our study include its 5-year follow-up and the use of a validated tool to measure QoL and patient reported outcomes. Study limitations are partly attributable to its nonrandomized design and the potential for recall bias to have impacted our findings. In addition, our sample size was small, limiting power for our comparisons. Other limitations of the study originated from real-world treatment pattens and choices; the disparity in group sizes may have limited statistical power and our ability to identify differences in outcomes and should be taken into account when interpreting the results. In our cohort, patients undergoing BO were older than those treated with GnRH which also reflects real-life patient preferences. While age and clinical stage varied between treatment groups, after adjustment for these variables, there were no statistically significant differences between treatment groups. However, they might be considered as confounders impacting both QoL reporting despite statistical adjustment for age and stage (Supplement Table S2). This was a single-institution study, impacting generalizability. While our study provides significant insights into the QoL outcomes during the initial 5 years, the long-term effects of BO and GnRH deserve further additional research.

We studied a significant gap in the literature by comparing long-term quality of life and oncologic outcomes between bilateral oophorectomy and GnRH in premenopausal women with hormone receptor-positive breast cancer who do not have a BRCA mutation. Although our primary objective was to evaluate quality of life, we also analyzed oncologic outcomes and observed that recurrence and survival rates were similar between the bilateral oophorectomy and GnRH cohorts, findings that they align with previous publications [9,40]. Our findings add to the current literature that both OFS strategies yield similar long-term quality of life outcomes, with no evident advantage of one method over the other. Our results highlight the importance of individualized decision-making that takes into account the patient’s age, preferences, and tolerance to treatment-related adverse effects. Further research should focus on optimizing survivorship care by integrating targeted interventions to address QoL concerns and mitigate long-term risks associated with OFS and GnRH.

## Data Availability

Data sharing is governed by institutional policies designed to protect privacy and other forms of sensitive information. Any reasonable request for access to data held by the Mayo Clinic will be carefully evaluated by the appropriate institutional authorities in accordance with applicable regulations, ethical standards, and internal protocols.

## Supplementary Materials

The following supporting information can be downloaded at https://www.mdpi.com/article/10.3390/cancers17172916/s1, Figure S1: Kaplan–Meier curves of disease-free survival comparing patients treated with bilateral oophorectomy versus gonadotropin-releasing hormone agonist; Table S1: Mean PROMIS-10 Mental and Physical Health T-scores at baseline and annually up to 5 years after breast cancer diagnosis, stratified by treatment group versus gonadotropin-releasing hormone agonist; Table S2: Age- and stage-adjusted longitudinal regression results for PROMIS-10 outcomes (linear mixed models). Estimates reflect fixed effects; values shown are estimates, 95% confidence intervals, and *p*-values.
