# Supplementary material for: A Comparative Study of Quality of Life and Oncologic Outcomes in Premenopausal Women with Hormone Receptor-Positive Breast Cancer: Bilateral Oophorectomy vs. Gonadotropin-Releasing Hormone Agonist Therapy
Source: Cancers (Basel). 2025 Sep 5;17(17):2916. doi: 10.3390/cancers17172916 (PMC12427670; doi:10.3390/cancers17172916)
Supplement: Supplementary file 1 [file cancers-17-02916-s001.zip › cancers-3779388-supplementary.pdf]

Supplement

Figure S1 Kaplan–Meier curves of disease-free survival comparing patients treated with bilateral oophorectomy versus gonadotropin-releasing hormone agonist.

Recurrence in BO group was lower but the difference between groups were statistically non-significant in 5 year follow-up. Time is given in years.

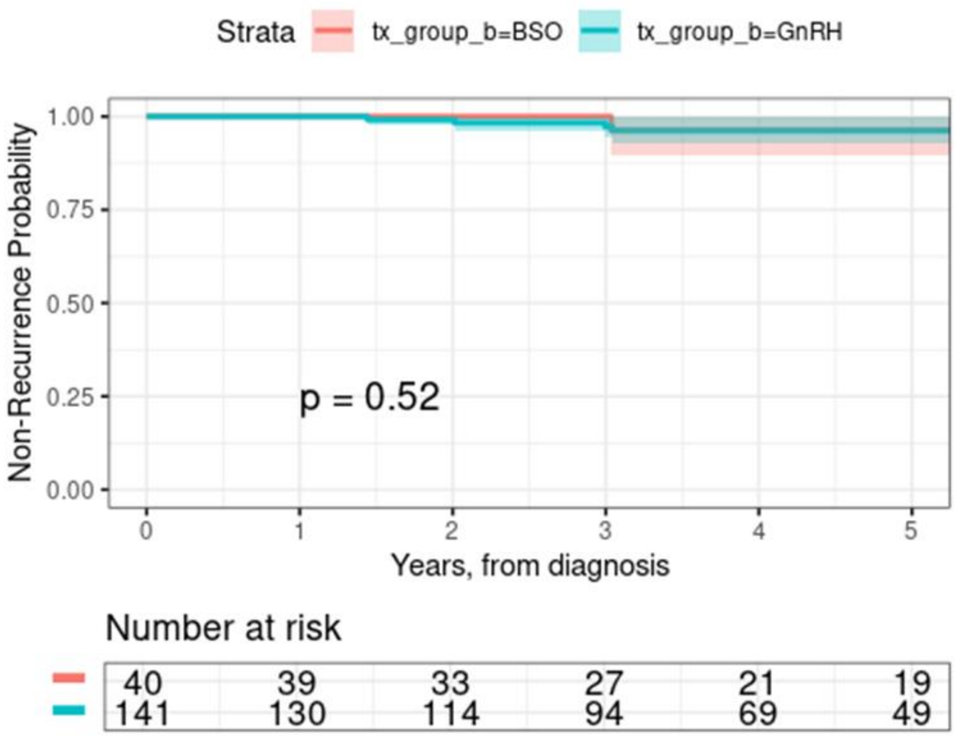

Estimated Marginal Means for Physical and Mental PROMIS-10 T-Scores

Table S1 Mean PROMIS-10 Mental and Physical Health T-scores at baseline and annually up to 5 years after breast cancer diagnosis, stratified by treatment group versus gonadotropin-releasing hormone agonist

Mental T-Score

Treatment Group	Time	Mean Score	lower.CL	upper.CL
factor	factor	numeric	numeric	numeric
BSO	Baseline	51.8	49.6	54.0
GnRH	Baseline	49.9	48.7	51.1
BSO	Y1	46.4	44.3	48.6
GnRH	Y1	46.4	45.2	47.6
BSO	Y2	50.7	48.2	53.3
GnRH	Y2	48.5	47.0	49.9
BSO	Y3	50.5	48.0	53.0
GnRH	Y3	48.7	47.4	50.1
BSO	Y4	50.4	47.8	53.1
GnRH	Y4	51.4	49.9	52.9
BSO	Y5	51.8	48.6	55.0
GnRH	Y5	52.6	50.5	54.7

n: 12

Physical T-Score

Treatment Group	Time	Mean Score	lower.CL	upper.CL
factor	factor	numeric	numeric	numeric
BSO	Baseline	50.9	48.7	53.2
GnRH	Baseline	50.6	49.4	51.8
BSO	Y1	50.4	48.2	52.6
GnRH	Y1	50.6	49.4	51.8
BSO	Y2	51.1	48.6	53.6
GnRH	Y2	51.8	50.5	53.2
BSO	Y3	50.8	48.4	53.3
GnRH	Y3	52.2	50.9	53.5
BSO	Y4	51.5	48.5	54.4
GnRH	Y4	50.8	49.0	52.6
BSO	Y5	51.3	47.6	54.9
GnRH	Y5	52.0	49.4	54.7

n: 12

Adjusted Analysis for Age and Stage

In models adjusted for **age** and **stage**, treatment group (BSO vs GnRH) was **not associated** with differences in PROMIS-10 global, physical, or mental scores over time; **older age** and **higher stage (especially stage III)** were associated with **modestly worse physical functioning and/or more pain**, while mental and social domains showed minimal age/stage effects.

Table S2. Age- and stage-adjusted longitudinal regression results for PROMIS-10 outcomes (linear mixed models). Estimates reflect fixed effects; values shown are estimates, 95% confidence intervals, and p-values.

Fixed-effect estimates are shown with 95% confidence intervals and p-values.

Outcome	Predictor	Estimate	CI Lower	CI Upper	p-value
**In general, would you say your health is:**	(Intercept)	4.3	3.7	5.0	**<0.0001**
**In general, would you say your health is:**	tx_group_bGnRH	0.0	-0.3	0.2	0.7505
**In general, would you say your health is:**	Time	0.0	0.0	0.1	0.0545
**In general, would you say your health is:**	clin_overallstageStage II	-0.2	-0.4	0.1	0.1327
**In general, would you say your health is:**	clin_overallstageStage III	-0.2	-0.5	0.1	0.2188
**In general, would you say your health is:**	age	0.0	0.0	0.0	**0.0241**
**In general, would you say your quality of life is:**	(Intercept)	4.6	4.0	5.3	**<0.0001**
**In general, would you say**	tx_group_bGnRH	-0.1	-0.4	0.1	0.2981

**your quality of life is:**					
**In general, would you say your quality of life is:**	Time	0.0	0.0	0.0	0.9816
**In general, would you say your quality of life is:**	clin_overallstageStage II	-0.1	-0.4	0.1	0.1960
**In general, would you say your quality of life is:**	clin_overallstageStage III	-0.2	-0.5	0.0	0.0874
**In general, would you say your quality of life is:**	age	0.0	0.0	0.0	**0.0426**
**In general, how would you rate your physical health?**	(Intercept)	4.4	3.7	5.1	**<0.0001**
**In general, how would you rate your physical health?**	tx_group_bGnRH	0.0	-0.2	0.3	0.8096
**In general, how would you rate your physical health?**	Time	0.0	0.0	0.0	0.4361
**In general, how would you rate your physical health?**	clin_overallstageStage II	-0.3	-0.6	-0.1	**0.0081**
**In general, how would**	clin_overallstageStage III	-0.3	-0.6	0.0	**0.0495**

**you rate your physical health?**					
**In general, how would you rate your physical health?**	age	0.0	0.0	0.0	**0.0075**
**In general, how would you rate your mental health, including your mood and your ability to think?**	(Intercept)	3.5	2.7	4.2	**<0.0001**
**In general, how would you rate your mental health, including your mood and your ability to think?**	tx_group_bGnRH	-0.1	-0.3	0.2	0.5474
**In general, how would you rate your mental health, including your mood and your ability to think?**	Time	0.0	0.0	0.0	0.7703
**In general, how would you rate your mental health, including your**	clin_overallstageStage II	0.0	-0.2	0.2	0.9979

**mood and your ability to think?**					
**In general, how would you rate your mental health, including your mood and your ability to think?**	clin_overallstageStage III	-0.1	-0.4	0.2	0.5204
**In general, how would you rate your mental health, including your mood and your ability to think?**	age	0.0	0.0	0.0	0.7603
**In general, how would you rate your satisfaction with your social activities and relationships?**	(Intercept)	4.1	3.4	4.9	**<0.0001**
**In general, how would you rate your satisfaction with your social activities and relationships?**	tx_group_bGnRH	-0.1	-0.4	0.2	0.4733
**In general, how would you rate your satisfaction**	Time	0.0	0.0	0.1	0.3355

**with your social activities and relationships?**					
**In general, how would you rate your satisfaction with your social activities and relationships?**	clin_overallstageStage II	0.0	-0.3	0.2	0.8561
**In general, how would you rate your satisfaction with your social activities and relationships?**	clin_overallstageStage III	-0.2	-0.5	0.2	0.3172
**In general, how would you rate your satisfaction with your social activities and relationships?**	age	0.0	0.0	0.0	0.1956
**In general, please rate how well you carry out your usual social activities and roles.**	(Intercept)	4.4	3.7	5.0	**<0.0001**
**In general, please rate how well you carry out your usual social**	tx_group_bGnRH	0.0	-0.3	0.2	0.8858

**activities and roles.**					
**In general, please rate how well you carry out your usual social activities and roles.**	Time	0.1	0.0	0.1	**0.0025**
**In general, please rate how well you carry out your usual social activities and roles.**	clin_overallstageStage II	-0.1	-0.3	0.1	0.2846
**In general, please rate how well you carry out your usual social activities and roles.**	clin_overallstageStage III	-0.2	-0.5	0.1	0.1150
**In general, please rate how well you carry out your usual social activities and roles.**	age	0.0	0.0	0.0	0.0520
**To what extent are you able to carry out your everyday physical activities such as walking, climbing stairs,**	(Intercept)	5.3	4.8	5.8	**<0.0001**

**carrying groceries, or moving a chair?**					
**To what extent are you able to carry out your everyday physical activities such as walking, climbing stairs, carrying groceries, or moving a chair?**	tx_group_bGnRH	-0.1	-0.2	0.1	0.5205
**To what extent are you able to carry out your everyday physical activities such as walking, climbing stairs, carrying groceries, or moving a chair?**	Time	0.0	0.0	0.0	0.1976
**To what extent are you able to carry out your everyday physical activities**	clin_overallstageStage II	-0.2	-0.3	0.0	0.0527

**such as walking, climbing stairs, carrying groceries, or moving a chair?**					
**To what extent are you able to carry out your everyday physical activities such as walking, climbing stairs, carrying groceries, or moving a chair?**	clin_overallstageStage III	-0.3	-0.5	-0.1	**0.0134**
**To what extent are you able to carry out your everyday physical activities such as walking, climbing stairs, carrying groceries, or moving a chair?**	age	0.0	0.0	0.0	**0.0355**
**In the past 7 days, how often have**	(Intercept)	3.0	2.4	3.5	**<0.0001**

**you been bothered by emotional problems such as feeling anxious, depressed, or irritable?**					
**In the past 7 days, how often have you been bothered by emotional problems such as feeling anxious, depressed, or irritable?**	tx_group_bGnRH	0.0	-0.2	0.2	0.9657
**In the past 7 days, how often have you been bothered by emotional problems such as feeling anxious, depressed, or irritable?**	Time	0.2	0.1	0.2	**<0.0001**
**In the past 7 days, how often have you been bothered by emotional problems such as**	clin_overallstageStage II	-0.2	-0.4	0.0	**0.0432**

**feeling anxious, depressed, or irritable?**					
**In the past 7 days, how often have you been bothered by emotional problems such as feeling anxious, depressed, or irritable?**	clin_overallstageStage III	-0.1	-0.3	0.2	0.4800
**In the past 7 days, how often have you been bothered by emotional problems such as feeling anxious, depressed, or irritable?**	age	0.0	0.0	0.0	0.8015
**In the past 7 days, how would you rate your fatigue on average?**	(Intercept)	4.2	3.6	4.8	**<0.0001**
**In the past 7 days, how would you rate your fatigue on average?**	tx_group_bGnRH	0.0	-0.2	0.2	0.9877

**In the past 7 days, how would you rate your fatigue on average?**	Time	0.1	0.0	0.1	**<0.0001**
**In the past 7 days, how would you rate your fatigue on average?**	clin_overallstageStage II	-0.1	-0.3	0.1	0.4543
**In the past 7 days, how would you rate your fatigue on average?**	clin_overallstageStage III	-0.1	-0.4	0.1	0.3534
**In the past 7 days, how would you rate your fatigue on average?**	age	0.0	0.0	0.0	0.0508
**In the past 7 days, how would you rate your pain on average? (0 - None, 10 - Worst Imaginable)**	(Intercept)	-0.3	-1.9	1.4	0.7664
**In the past 7 days, how would you rate your pain on average? (0 - None, 10 - Worst Imaginable)**	tx_group_bGnRH	-0.2	-0.8	0.4	0.5747

**In the past 7 days, how would you rate your pain on average? (0 - None, 10 - Worst Imaginable)**	Time	0.0	-0.1	0.0	0.2415
**In the past 7 days, how would you rate your pain on average? (0 - None, 10 - Worst Imaginable)**	clin_overallstageStage II	0.1	-0.4	0.7	0.6922
**In the past 7 days, how would you rate your pain on average? (0 - None, 10 - Worst Imaginable)**	clin_overallstageStage III	0.4	-0.3	1.1	0.3031
**In the past 7 days, how would you rate your pain on average? (0 - None, 10 - Worst Imaginable)**	age	0.1	0.0	0.1	**0.0026**
**PROMIS 10 - Global Raw Score**	(Intercept)	42.8	37.8	47.7	**<0.0001**
**PROMIS 10 - Global Raw Score**	tx_group_bGnRH	-0.5	-2.3	1.3	0.6007

**PROMIS 10 - Global Raw Score**	Time	0.3	0.1	0.5	**0.0148**
**PROMIS 10 - Global Raw Score**	clin_overallstageStage II	-1.2	-2.8	0.5	0.1697
**PROMIS 10 - Global Raw Score**	clin_overallstageStage III	-1.8	-3.9	0.3	0.0989
**PROMIS 10 - Global Raw Score**	age	-0.1	-0.2	0.0	**0.0302**
**PROMIS 10 - Mental Health T-Score**	(Intercept)	52.0	46.2	57.9	**<0.0001**
**PROMIS 10 - Mental Health T-Score**	tx_group_bGnRH	-0.9	-3.1	1.2	0.3789
**PROMIS 10 - Mental Health T-Score**	Time	0.5	0.2	0.8	**0.0002**
**PROMIS 10 - Mental Health T-Score**	clin_overallstageStage II	-1.0	-2.9	1.0	0.3318
**PROMIS 10 - Mental Health T-Score**	clin_overallstageStage III	-1.5	-3.9	1.0	0.2381
**PROMIS 10 - Mental Health T-Score**	age	-0.1	-0.2	0.1	0.3243
**PROMIS 10 - Physical Health T-Score**	(Intercept)	60.9	54.7	67.2	**<0.0001**
**PROMIS 10 - Physical Health T-Score**	tx_group_bGnRH	-0.1	-2.4	2.1	0.9115
**PROMIS 10 - Physical**	Time	0.3	0.0	0.6	**0.0223**

**Health T-Score**					
**PROMIS 10 - Physical Health T-Score**	clin_overallstageStage II	-1.8	-3.9	0.3	0.0919
**PROMIS 10 - Physical Health T-Score**	clin_overallstageStage III	-2.9	-5.5	-0.3	**0.0315**
**PROMIS 10 - Physical Health T-Score**	age	-0.2	-0.3	-0.1	**0.0011**
**PROMIS 10 - Mental Raw Score**	(Intercept)	15.3	13.0	17.5	**<0.0001**
**PROMIS 10 - Mental Raw Score**	tx_group_bGnRH	-0.3	-1.1	0.5	0.4123
**PROMIS 10 - Mental Raw Score**	Time	0.2	0.1	0.3	**0.0004**
**PROMIS 10 - Mental Raw Score**	clin_overallstageStage II	-0.4	-1.1	0.4	0.3500
**PROMIS 10 - Mental Raw Score**	clin_overallstageStage III	-0.6	-1.5	0.4	0.2363
**PROMIS 10 - Mental Raw Score**	age	0.0	-0.1	0.0	0.3479
**PROMIS 10 - Physical Raw Score**	(Intercept)	18.9	16.9	20.9	**<0.0001**
**PROMIS 10 - Physical Raw Score**	tx_group_bGnRH	0.0	-0.7	0.7	0.9698

**PROMIS 10 - Physical Raw Score**	Time	0.1	0.0	0.2	**0.0376**
**PROMIS 10 - Physical Raw Score**	clin_overallstageStage II	-0.6	-1.3	0.1	0.0705
**PROMIS 10 - Physical Raw Score**	clin_overallstageStage III	-0.9	-1.7	-0.1	**0.0369**
**PROMIS 10 - Physical Raw Score**	age	-0.1	-0.1	0.0	**0.0017**

PROMIS-10 Questions

	Please respond to each item by marking **one box per row**	Excellent	Very good	Good	Fair	Poor
Global 01	In general, would you say your health is:	☐ 5	☐ 4	☐ 3	☐ 2	☐ 1
Global 02	In general, would you say your quality of life is:	☐ 5	☐ 4	☐ 3	☐ 2	☐ 1
Global 03	In general, how would you rate your physical health?	☐ 5	☐ 4	☐ 3	☐ 2	☐ 1
Global 04	In general, how would you rate your mental health, including your mood and your ability to think?	☐ 5	☐ 4	☐ 3	☐ 2	☐ 1
Global 05	In general, how would you rate your satisfaction with your social activities and relationships?	☐ 5	☐ 4	☐ 3	☐ 2	☐ 1
Global 09	In general, please rate how well you carry out your usual social activities and roles. (This includes activities at home, at work and in your community, and responsibilities as a parent, child, spouse, employee, friend, etc.)	☐ 5	☐ 4	☐ 3	☐ 2	☐ 1
		**Completely**	**Mostly**	**Moderately**	**A Little**	**Not At All**
Global 06	To what extent are you able to carry out your everyday physical activities such as walking, climbing stairs, carrying groceries, or moving a chair?	☐ 5	☐ 4	☐ 3	☐ 2	☐ 1
	**In the past 7 days**	**Never**	**Rarely**	**Sometimes**	**Often**	**Always**
Global 10	How often have you been bothered by emotional problems such as feeling anxious, depressed or irritable?	☐ 5	☐ 4	☐ 3	☐ 2	☐ 1
		**None**	**Mild**	**Moderate**	**Severe**	**Very Severe**
Global 08	How would you rate your fatigue on average?	☐ 5	☐ 4	☐ 3	☐ 2	☐ 1
Global 07	How would you rate your pain on average?	☐ 0 ☐ 1 ☐ 2 ☐ 3 ☐ 4 ☐ 5 ☐ 6 ☐ 7 ☐ 8 ☐ 9 ☐ 10<br>0 = No Pain; 10 = Worst Imaginable Pain				

Scoring:
Re-code Global07. The recoded score ranges from 1 to 5.
(0 No pain =**5**; 1, 2, or 3 =**4**; 4, 5, or 6 =**3**; 7, 8, or 9 =**2**; 10 worst pain imaginable =**1**)

After recoding, the
Global Physical Health score = SUM responses to G03 + G06 + G07 + G08.
Global Mental Health score = SUM G02 + G04 + G05 + Global10.

Responses: Excellent, Very Good, Good, Fair, Poor
